# Direct Determination of the Site of Addition of Glucosyl Units to Maltooligosaccharide Acceptors Catalyzed by Maize Starch Synthase I

**DOI:** 10.3389/fpls.2018.01252

**Published:** 2018-08-31

**Authors:** Ying Xie, Adam W. Barb, Tracie A. Hennen-Bierwagen, Alan M. Myers

**Affiliations:** ^1^Roy J. Carver Department of Biochemistry, Biophysics, and Molecular Biology, Iowa State University, Ames, IA, United States; ^2^College of Agronomy, Sichuan Agricultural University, Chengdu, China

**Keywords:** maize, starch synthase, enzymology, starch, amylopectin metabolism

## Abstract

Starch synthase (SS) (ADP-glucose:1,4-α-D-glucan 4-α-D-glucosyltransferase) elongates α-(1→4)-linked linear glucans within plastids to generate the storage polymers that constitute starch granules. Multiple SS classes are conserved throughout the plant kingdom, indicating that each provides a unique function responsible for evolutionary selection. Evidence has been presented arguing for addition of glucosyl units from the ADPglucose donor to either the reducing end or the non-reducing end of the acceptor substrate, although until recently direct evidence addressing this question was not available. Characterization of newly incorporated glucosyl units determined that recombinant maize (*Zea mays* L.) SSIIa elongates its substrates at the non-reducing end. However, the possibility remained that other SSs might utilize distinct mechanisms, and that one or more of the conserved enzyme classes could elongate acceptors at the reducing end. This study characterized the reaction mechanism of recombinant maize SSI regarding its addition site. Newly incorporated residues were labeled with ^13^C, and reducing ends of the elongation products were labeled by chemical derivitization. Electrospray ionization-tandem mass spectroscopy traced the two parameters, i.e., the newly added residue and the reducing end. The results determined that SSI elongates glucans at the non-reducing end. The study also confirmed previous findings showing recombinant SSI can generate glucans of at least 25 units, that it is active using acceptors as short as maltotriose, that recombinant forms of the enzyme absolutely require an acceptor for activity, and that it is not saturable with maltooligosaccharide acceptor substrates.

## Introduction

Starch synthases (ADP-glucose:1,4-α-D-glucan 4-α-D-glucosyltransferase) (SS) fulfill a critical function in our biosphere by converting photosynthetically-reduced carbon into storage polymers that subsequently provide chemical energy necessary for plant life cycles and indirectly for animal life cycles as well. These enzymes catalyze the transfer of glucosyl moieties from ADP-glucose (ADPGlc) donors to α-(1→4)-linked glucan polymer acceptors, elongating the polymers by one unit each reaction cycle (Keeling and Myers, 2010). Products of cumulative SS activity, amylopectin and amylose, also include α-(1→6) branch linkages introduced by starch branching enzymes (SBEs). Selected hydrolysis of a subset of branch linkages by starch debranching enzymes (DBEs) is also involved in the amylopectin biosynthetic mechanism (Hennen-Bierwagen et al., 2012). Together this enzyme system generates polymers that adopt a semi-crystalline structure within starch granules, and such higher order organization enables long term photosynthate storage (Jeon et al., 2010). Thus, architectural specificity of SS action, in addition to the chemical reaction itself, is responsible for the important functional role of these enzymes.

Architectural organization of glucan polymers in starch granules involves multiple SS conserved in apparently all chloroplast-containing species, i.e., green algae and land plants. Genes encoding five SS classes are present in each genome examined to date, designated GBSS, SSI, SSII, SSIII, and SSIV (Deschamps et al., 2008; Hennen-Bierwagen and Myers, 2013). In some species gene duplication has resulted in multiple isoforms within a class. Strict evolutionary conservation indicates each SS class likely provides a selected function, some of which are relatively well-understood and others that remain to be determined. GBSS generates long linear polymers of several 100 residues that make up amylose, and SSIV appears to have a particular function in initiating starch granule formation (Szydlowski et al., 2009). SSI, SSII, and SSIII generate linear chains that crystallize to form clusters within amylopectin, although their specific functions and how they impart them at the molecular level are not known (for review see Zeeman et al., 2010; Nakamura, 2015). Genetic analyses revealing effects of eliminating specific SS enzymes have led to models suggesting SSI elongates linear chains created by SBE action, starting at degree of polymerization (DP) 6 or 7, to approximately DP12, then SSII elongates those chains further to a distribution centered about DP20 (Crofts et al., 2017). These chains then crystallize into clusters that are the basis of the higher order starch structures. SSIII is proposed from such studies to generate chains of DP30 and longer that connect clusters (Fujita et al., 2007; Zhu et al., 2013).

Such models, however, do not fully explain SS action *in vivo* nor the reason that each class is conserved. They are based primarily on findings that eliminating an SS changes the frequency of occurrence of a particular chain length, rather than eliminating formation of such chains. For example, as the general model indicates, loss of SSII in multiple species results in decreased abundance of approximately DP12 to DP20 chains (Zhang et al., 2004 and references therein). Chains in that length range still exist in SSII mutants, however, at approximately 80% of the normal level. Thus, there must be considerable overlap in the ability of other SS classes to create DP12–DP20 chains. Similarly, loss of SSI, in *Arabidopsis* leaf or rice endosperm causes decreased frequency of approximately DP8 to DP12, yet chains of those lengths are still present in the mutant amylopectin (Delvallé et al., 2005; Fujita et al., 2006; Crofts et al., 2017). Further, there is little evidence available indicating distinctions in substrate specificities between SS clases. *In vitro* studies with recombinant enzymes comparing properties of different SS classes found similar reaction rates of SSI and SSII toward linear glucans from DP2 to DP8, and that SSI is capable of elongating to DP40 and longer (Cuesta-Seijo et al., 2015). Thus, there is no obvious mechanistic reason that explains why SSI, SSII, and SSIII each are invariably retained throughout green plant evolution.

A fundamental question regarding SS mechanism is whether addition of glucosyl residues occurs at the reducing- or non-reducing end of the acceptor chain. A non-reducing end mechanism was long accepted in the literature, based on findings that newly incorporated labeled residues are released by exohydrolases known to proceed from that terminus of linear glucans (De Fekete et al., 1960; Leloir et al., 1961; Recondo and Leloir, 1961). Such a mechanism would enable rapid growth of branched amylopectin precursors, because such molecules have only a single reducing end compared to 1000s of non-reducing ends. More recently, reducing end addition by SSs was proposed based on the observation that newly incorporated glucosyl residues can be chemically derivatized in reactions that require free aldehyde groups (Mukerjea et al., 2002; Mukerjea and Robyt, 2005a,b, 2012).

Direct determination of the site of addition of new glucosyl residues has been described in only one instance. Recombinant SSIIa from maize was used to elongate maltohexaose, using ADPGlc containing uniformly ^13^C-labeled glucosyl units (ADP-[^13^C_U_]-Glc). Newly incorporated residues could thus be distinguished from pre-existing residues by a mass difference of 6 Da. After the polymers were elongated by SSIIa, they were labeled by reductive amination with *N*-naphthyl-ethylene diamine (*N*-EDA), adding a defined mass label specifically to the reducing end residue. Electrospray ionization-tandem mass spectroscopy (ESI-MS/MS) detected addition of [^13^C_U_]-Glc units, then determined the molecular mass of the derivatized reducing end residue that was covalently labeled with the N-EDA tag. In these experiments, N-EDA mass label was attached uniquely to ^12^C_U_-glucose, meaning only pre-existing residues within maltohexaose, not newly added glucosyl units from the enzymatic reaction, were located at the reducing end (Larson et al., 2016). This provides direct evidence for non-reducing end addition by maize SSIIa. That conclusion was confirmed by ^13^C-NMR analyses that distinguished between free anomeric carbons and those bound in α-(1→4) glycoside bonds. Anomeric carbons in newly incorporated residues were found to be glycoside-bound, not free, thus confirming non-reducing end addition (Larson et al., 2016).

The possibility remains that distinctions between SS classes could include different reaction mechanisms, and that SSs other than maize SSIIa could elongate glucan polymers at the reducing end. This is pertinent because the experiments supporting a reducing end mechanism used starch granules as a SS source to catalyze addition of new residues (Mukerjea et al., 2002; Mukerjea and Robyt, 2005a), and these contain multiple enzymes including SSI, SSII, and GBSS (Grimaud et al., 2008). To test whether non-reducing end addition is universal among SS classes, the current study characterized addition products formed by recombinant maize SSI. [^13^C_U_-Glc] labeling and product characterization by ESI-MS/MS revealed exclusive elongation at the non-reducing end. Thus, as expected from structural similarities among the enzymes, non-reducing end addition is highly likely to be universal among all SS classes.

## Materials and Methods

### Recombinant SSI Expression

Recombinant maize SSI was expressed from plasmid pET29-NStagSSI (Huang et al., 2016). The pET-29 vector backbone provides the phage T7 promoter, and an *E. coli* ribosome binding site prior to the coding region. The expressed protein, designated NS-SSI, contains the 15 amino acid S-tag sequence at its N terminus followed by the mature maize SSI coding sequence. Expression of recombinant protein in *E. coli* Rosetta (DE3) pLys by induction of phage T7 polymerase with IPTG was as previously described. Briefly, cell pellets from 50 mL of induced culture were suspended in buffer A (20 mM Tris-HCl, pH 7.5, 150 mM NaCl) supplemented with 0.1 mg/mL lysozyme, 5 mM DTT, 1 mM PMSF. Cells were lysed by sonication, then lysates were cleared by centrifugation and filtration through a 0.45 μm syringe filter. NS-SSI was purified from cleared lysates by affinity to S-protein agarose equilibrated in buffer A. After binding, matrix with bound NS-SSI, referred to as “SSI beads,” was washed extensively in buffer A and stored on ice. SSI beads were used directly as the catalyst in enzymatic reactions, within 2 days of their preparation.

Protein concentration on SSI beads was estimated after incubating 10 μL samples of a 1:1 bead:buffer A slurry (v:v) with 20 μL of 8 M urea to release bound ligand. Beads were removed by centrifugation in a microfuge, then the A_280_ of the supernatant was measured using a NanoDrop spectrophotomer. Protein concentration was calculated from the molar extinction coefficient of 112,760 mol^-1^ cm^-1^ predicted for the NS-SSI amino acid sequence. Typical purifications yielded approximately 15 μg protein per 2 μL of SSI bead slurry.

### Enzyme Assays

Reactions were incubated at 30°C in 100 μL final volume of 100 mM bicine-NaOH, pH 8.0, 5 mM EDTA, 0.5 mg/mL BSA, 5 mM DTT. Rate measurements were from assays including 2 μL of SSI bead slurry (approximately 15 μg NS-SSI). Preliminary analyses with excess ADPGlc and glycogen determined that such reactions were rate-limited for enzyme. For kinetic analyses, glycogen concentration was constant at 10 mg/mL and ADPGlc concentration varied from 0.2 to 2.0 mM. Rate measurements using maltooligosaccharides were from reactions containing 2 mM ADPGlc, with acceptor concentration varying from 10 to 80 mM. Reactions were initiated by addition of SSI beads and terminated at various times up to 30 min by boiling for 1 min. Beads were removed by brief centrifugation and ADP product in 5 μL of the supernatant was quantified using the ADP Glo Kinase Assay Kit (Promega No. V6930) as described previously (Huang et al., 2016; Larson et al., 2016).

Other reactions were carried out in conditions of enzyme excess, using [^13^C_U_]-ADPGlc prepared as previously described (Larson et al., 2016). These “enzyme excess” reactions varied from the kinetic analyses in that they contained 50 μL of SSI bead slurry, 4 mM [^13^C_U_]-ADPGlc, and 2 mM maltooligosaccharide, either maltotriose (DP3), maltotetraose (DP4), or maltopentaose (DP5). These reactions were incubated from 1 to 24 h.

### Product Analysis

Products of enzyme excess reactions were analyzed by TLC, high pressure anion exchange chromatography with pulsed amperometric detection (HPAEC-PAD), and ESI-MS/MS. The TLC method was described previously (Mukerjea et al., 2010; Larson et al., 2016). For HPAEC-PAD analysis, terminated reaction supernatant was diluted 1:20 into H_2_O and filtered through a 0.2 μm syringe filter, then 25 μL was injected into a Dionex IC-5000 chromatography system. Separation was on a Dionex CarboPac PA-100 4 mm × 250 mm column with a CarboPac PA-100 4 mm × 50 mm guard column at a flow rate of 1 mL/min. Elution was in 0.1 M NaOH for 4 min followed by a 40 min gradient of 0–0.4 M sodium acetate in 0.1 M NaOH. For ESI-MS/MS analysis, reaction products were first derivatized at the reducing end by reductive amination with N-EDA. Methods and instrumentation for derivatization and subsequent ESI-MS/MS analysis were described previously (Larson et al., 2016). MS1 spectra were averaged over fractions eluting from 9.2 to 9.4 min. MS2 spectra were averaged over all fractions.

## Results

### Purification and Characterization of Recombinant SSI

**FIGURE 1:**
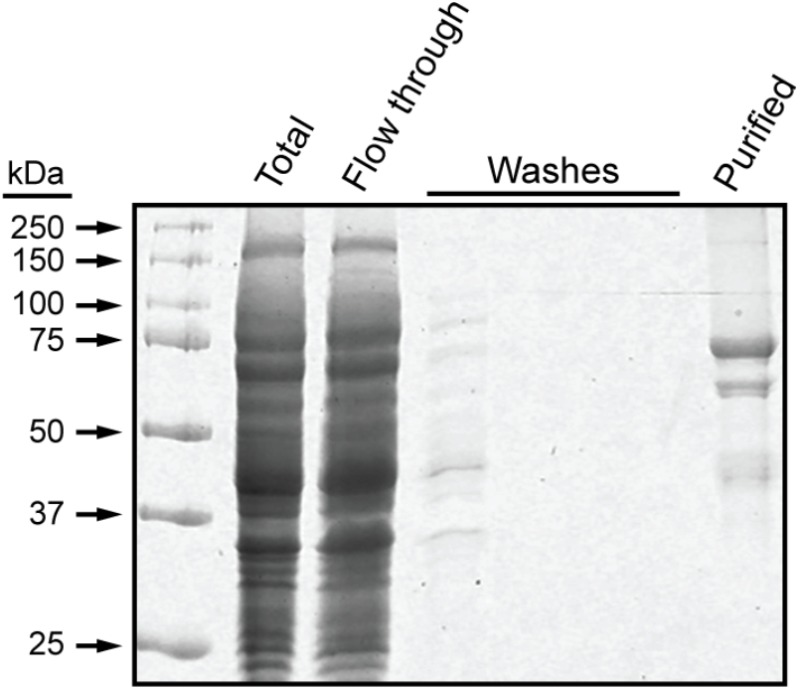
NS-SSI purification. NS-SSI was purified from total soluble *E. coli* extract by binding to S-protein agarose. Proteins in each fraction were visualized by SDS-PAGE and Coomassie blue staining. The purified fraction was analyzed after boiling of the affinity matrix in SDS-PAGE loading buffer.

These experiments utilized a recombinant form of maize SSI that contains the 15 amino acid S-tag sequence at the N terminus, as previously described (Huang et al., 2016). Residues 39–640 of full-length SSI were included, corresponding precisely to those present in mature protein following transit peptide removal during import into amyloplasts (Knight et al., 1998). The recombinant protein, termed NS-SSI, was expressed in *E. coli* and affinity purified from the soluble phase by binding to S-protein agarose. The purified fraction contained a major protein of approximately 70 kDa, matching the predicted molecular mass of NS-SSI (**Figure 1**). A minor contaminant of approximately 45 kDa was shown to be a fragment of SSI, by immunoblot using SSI-specific antiserum (data not shown). An unidentified minor contaminant protein of approximately 60 kDa was also present in the purified fraction.

**FIGURE 2:**
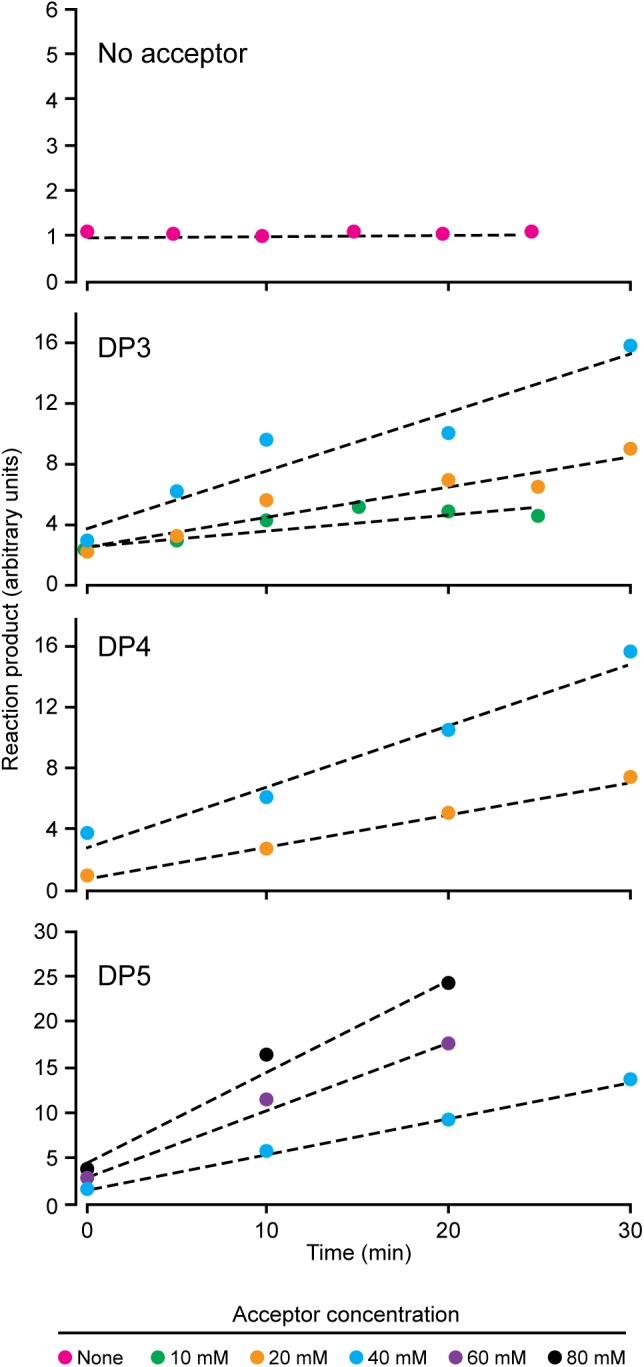
Reaction velocity with variable concentration of acceptor substrate. Reactions were saturated for ADPGlc and limited for NS-SSI. Reaction rates were determined by measuring molecules of ADP released at each time point.

NS-SSI bound to S-agarose was characterized to ensure it exhibited expected, reproducible enzymatic characteristics. Assays in the presence of ADPGlc donor and various maltooligosaccharide or polysaccharide acceptors were initiated by addition of S-agarose-NS-SSI. Preliminary experiments ensured that reaction rates were linear with bead bed volume. Aliquots of the reaction mixture were removed at various times, catalysis was terminated by boiling, S agarose was removed by centrifugation, and ADP product was quantified by a commercial chemiluminescence assay. Kinetic analyses revealed the Km for ADPGlc, using either maltooligosaccharide or glycogen as the acceptor, to be approximately 0.5 mM. This value approximates the Km previously described for recombinant SSI from either maize or barley (Cuesta-Seijo et al., 2015; Huang et al., 2016). Holding ADPGlc concentration constant at 2 mM, approximately fourfold above the Km, allowed testing of rates with variable concentrations of linear glucan acceptors, specifically, maltotriose (DP3), maltotetraose (DP4), and maltopentaose (DP5) (**Figure 2**). NS-SSI was active using any of these acceptors as the initial substrate. However, saturation of enzyme-substrate complex was never attained with any of the three glucans, even with up to 80 mM acceptor in a reaction containing < 5 μM enzyme. Such non-Michaelis-Menten behavior was observed previously for recombinant maize SSIIa (Larson et al., 2016), and recombinant SSI, SSII, and SSIII from barley (Cuesta-Seijo et al., 2015). In these *in vitro* conditions, NS-SSI required an acceptor glucan for activity (**Figure 2**).

### Enzyme Saturation Reactions

**FIGURE 3:**
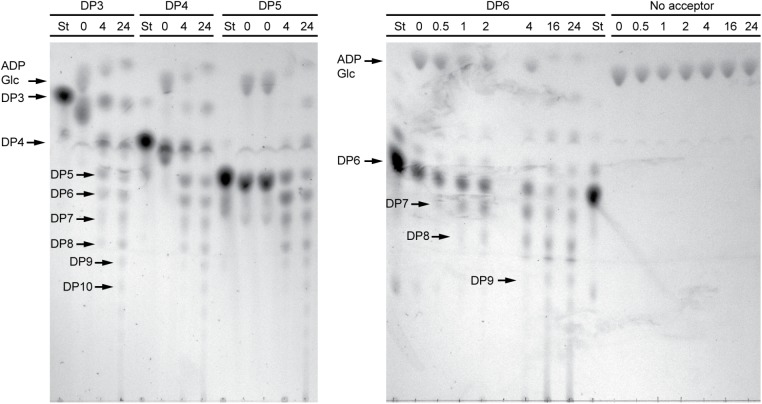
Thin layer chromatography (TLC) analysis of NS-SSI reaction products. Reactions were in enzyme excess conditions. The maltooligosaccharide acceptor used in each reaction, or lack therof, is indicated. Numbers indicate reaction times in hours. “St” indicates maltooligosaccharide standard for each acceptor.

In order to structurally analyze glucan products, reactions were run with excess enzyme to near complete depletion of ADPGlc, so that most of the acceptor substrate would be elongated. Product formation was observed by thin layer chromatography (TLC) (**Figure 3**). In these conditions NS-SSI was active on DP3, DP4, DP5, or DP6 to approximately the same extent. In each instance the majority of initial acceptor substrate molecules was converted to a mixture of longer polymers of increasing degrees of polymerization. Polymerization of glucosyl units was dependent on presence of an acceptor glucan, as shown by lack of higher molecular weight molecules when glucan was omitted from the reaction. Accordingly, ADPGlc was not consumed when incubated with NS-SSI in the absence of a glucan substrate.

**FIGURE 4:**
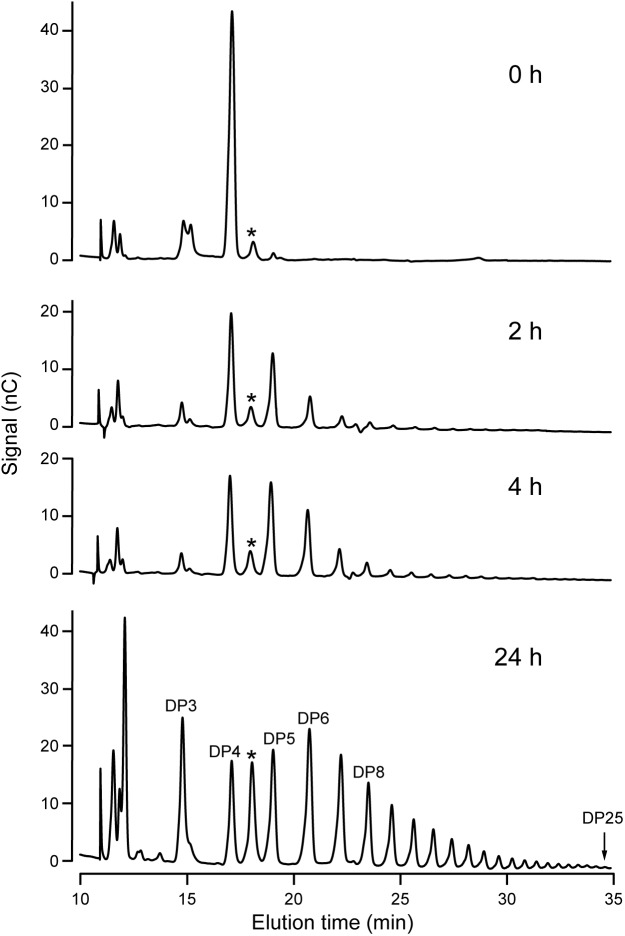
HPAEC-PAD analysis of NS-SSI reaction products. The initial acceptor substrate was DP4, and reactions were in conditions of enzyme excess. Reaction times are indicated. Products are identified based on standards. Asterisk indicates an unidentified peak in the reaction mixture.

Increased resolution of the products formed in the reaction was achieved using HPAEC-PAD. An example is shown using DP4 as the initial acceptor (**Figure 4**). Over a 24 h period, NS-SSI catalyzed formation of a range of linear chains extending to at least DP25. At the last time point, glucans shorter than the initial substrate, specifically DP2 and DP3, were prevalent within the mixture of products. Removal of glucosyl residues is expected to result from the reverse reaction catalyzed by SS that occurs as ADPGlc is consumed and the concentration of ADP product increases. Reversible SS activity was demonstrated previously for recombinant forms of *Arabidopsis* and barley SSI (Brust et al., 2013; Cuesta-Seijo et al., 2015). Alternatively, the trivial explanation that a contaminant in the S-protein agarose fraction bound to NS-SSI possesses glucan hydrolase activity, has not been ruled out.

### Structural Characterization of Newly Polymerized Linear Glucans

**FIGURE 5:**
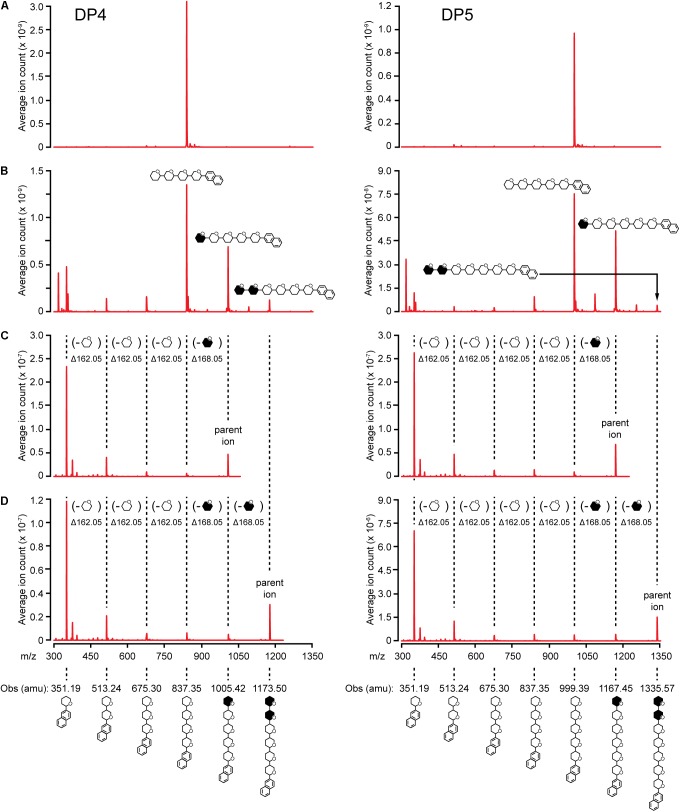
MS/MS analysis of NS-SSI reaction products. The initial acceptor substrate used in each column is indicated. Symbols indicate the molecule generating each peak, including glucosyl residues and N-EDA reducing end label. Open or closed hexagons represent [^12^C]- or [^13^C]-glucosyl units, respectively. **(A)** MS1 spectra of control reaction lacking enzyme. **(B)** MS1 spectra NS-SSI-catalyzed reaction products. **(C)** MS2 spectra of products elongated by one residue. **(D)** MS2 spectra of products elongated by two residues.

Elongation reactions catalyzed by NS-SSI in saturating conditions were performed using DP3, DP4, and DP5 with [^13^C_U_]-ADPGlc as the donor substrate, in order to distinguish between newly added glucosyl units and pre-existing residues of the acceptor. After 4 h of reaction in excess enzyme conditions and limiting [^13^C_U_]-ADPGlc, reducing ends of the glucan products were labeled by reductive amination with N-EDA. Molecules present in the labeling reactions were fractionated by reverse-phase chromatography, then subjected to electrospray ionization, and resulting ions were characterized by mass spectroscopy. Chromatographic fractions containing mass features matching the prediction for N-EDA-labeled initial substrate were identified. In reactions lacking enzyme these mass features were essentially pure (**Figure 5A**). Larger masses appeared in the spectra of reactions including NS-SSI that matched the prediction for N-EDA-labeled glucans elongated by one or two glucosyl units derived from [^13^C_U_]-ADPGlc (**Figure 5B**). Reaction products containing fewer glucosyl units than the starting acceptor substrate were also detected in MS1 spectra. These presumably result from the SSI reverse reaction, as was also observed by HPAEC-PAD analysis (**Figure 4**).

^13^C-labeled glucosyl units potentially could have been added at either the non-reducing or reducing end. Analysis of mass fragmentation patterns collected by selecting then fragmenting the parent ions distinguished between these possibilities. The parent ion derived from extension of the substrate by one unit was subjected to further ionizing radiation and the masses of the resultant fragmentation ions were determined. If a newly incorporated [^13^C]-labeled glucose residue were added at the reducing end, then derivatized with N-EDA, the molecular mass of the fragment containing one glucose unit would be 357.19 Da. Alternatively, if new incorporations occur at the non-reducing end, then N-EDA would be linked to an [^12^C]-glucosyl unit pre-existing in the acceptor substrate. In that instance the molecular mass of the fragment containing one [^12^C]-glucosyl unit would be 351.19 Da. Fragments of 357.19 Da were never observed in the second dimension mass spectra, whereas fragments of 351.19 were prominent. These data indicate the acceptor substrate was elongated at the non-reducing end(**Figures 5C,D**).

Further, the difference in mass between the parent ion and the second dimension fragment that had lost one glucosyl unit could be determined. The unit that is removed must be at the non-reducing end, because all fragments in this analysis retain the N-EDA moiety located at the reducing end. This mass difference was 168.05 Da, indicative of a newly incorporated residue. Taken together the data provide conclusive evidence that glucosyl units derived from ADP-Glc, and added to glucan acceptors by NS-SSI action, are located at the non-reducing end.

## Discussion

The current study adds to previous characterization of the site of addition of new glucosyl units to growing α(1→4)-linked glucan polymers as catalyzed by SS. Together the data demonstrate definitively that recombinant SSI and SSIIa from maize both add glucose units at the non-reducing end. These results are fully consistent with the known structure of *E. coli* glycogen synthase bound to maltooligosaccharide acceptor. Prokaryotic glycogen synthases and the catalytic domain of plant SSs are conserved at the amino acid level, and both exhibit the GT-B structural fold (Sheng et al., 2009a; Cuesta-Seijo et al., 2013). The *E. coli* enzyme has been co-crystallized with maltohexaose, and that structure reveals three bound glucosyl units with the non-reducing residue opposed to the ADPGlc donor substrate (Sheng et al., 2009b). Taken together these data definitively eliminate the proposed mechanism in which glucosyl units are added at the reducing end of the acceptor. Further, the fact that both SSI and SSII have now been shown to utilize non-reducing end mechanisms indicates that all of the five conserved classes of this enzyme likely act in the same way.

The reasons that five classes of SS are conserved in the plant kingdom remain to be determined. However, alternative mechanisms of glucose unit addition in the various enzyme classes is highly unlikely to be part of the explanation. The current study further confirms the previous finding that evolutionary selection of SSI does not relate to limits on the degree of polymerization of the products, as had been proposed based on indirect measurement of affinity to branched glucans with varying chain length distributions (Commuri and Keeling, 2001). HPAEC-PAD characterization of NS-SSI products starting from maltotetraose showed the enzyme is capable of producing glucans of at least DP25. Previous data with recombinant barley SSI showed it capable of generating product of DP40 or more (Cuesta-Seijo et al., 2015). Thus, the SSI class in general is not inherently restricted to production of chains of DP12 or less, as could be predicted from genetic analyses showing accumulation of such chains when SSII is absent. The reasons for these observed alterations in amylopectin structure in mutant plant tissues, therefore, likely are related to *in vivo* factors that are not reproduced in recombinant systems.

## Author Contributions

YX, TH-B, and AM performed the experiments and analyzed the data. TH-B, AB, and AM conceived the experiments and analyzed the data. All authors contributed to drafting the manuscript and approved the final version.

## Conflict of Interest Statement

The authors declare that the research was conducted in the absence of any commercial or financial relationships that could be construed as a potential conflict of interest. The reviewer IT and handling Editor declared their shared affiliation.
